# A Synthetic Bioactive Peptide Derived from the Asian Medicinal Plant *Acacia catechu* Binds to Dengue Virus and Inhibits Cell Entry

**DOI:** 10.3390/v12111267

**Published:** 2020-11-06

**Authors:** Aussara Panya, Nunghathai Sawasdee, Pucharee Songprakhon, Yingmanee Tragoolpua, Siriphorn Rotarayanont, Kiattawee Choowongkomon, Pa-thai Yenchitsomanus

**Affiliations:** 1 Department of Biology, Faculty of Science, Chiang Mai University, Chiang Mai 50200, Thailand; aussara.pan@cmu.ac.th (A.P.); yboony150@gmail.com (Y.T.); siriphorn.biocmu@gmail.com (S.R.); 2 Research Center in Bioresources for Agriculture, Industry and Medicine, Faculty of Science, Chiang Mai University, Chiang Mai 50200, Thailand; 3 Division of Molecular Medicine, Research Department, Faculty of Medicine Siriraj Hospital, Mahidol University, Bangkok 10700, Thailand; sawasdee111@gmail.com (N.S.); tay_pcr@hotmail.com (P.S.); 4 Department of Biochemistry, Faculty of Science, Kasetsart University, Bangkok 10900, Thailand; kiattawee.c@ku.th

**Keywords:** dengue virus, antiviral agent, peptide inhibitor, *Acacia catechu*, bioactive peptide

## Abstract

Dengue virus (DENV) infection has become a critically important globally prevalent infectious disease, especially in tropical and subtropical countries. Since neither currently exists, there is an urgent need for an effective vaccine to prevent, and a specific drug to treat DENV infection. Therapeutic peptides represent an attractive alternative for development into anti-DENV drugs due to their safety and their diverse biological and chemical properties. We recently reported novel bioactive peptides extracted from the Asian medicinal plant *Acacia catechu* that efficiently inhibited all four DENV serotypes. In this study, we investigated the anti-DENV activity of a synthetic bioactive peptide derived from this plant. The most effective peptide (designated Pep-RTYM) inhibited DENV infection with a half-maximal inhibition concentration value of 7.9 μM. Time-of-addition study demonstrated that Pep-RTYM interacted with DENV particles and inhibited cellular entry. Pep-RTYM at 50 μM significantly reduced DENV production in Vero-kidney epithelial cells about 1000-fold, but it could decrease the virus production in Huh7 hepatocyte cells approximately 40-fold. Binding of Pep-RTYM to DENV particles may prevent virus interaction with cellular receptor and subsequent virus entry. This finding suggests a potential role of Pep-RTYM in the development of a novel anti-DENV drug.

## 1. Introduction

Dengue virus (DENV), which is a member of the genus Flavivirus of the Flaviridae family, is an enveloped positive single-stranded RNA virus [1]. DENV infection is a critical health problem in tropical and subtropical countries, and it causes significant socio-economic burden. Over the past five decades, DENV has gone from being an infection that occurred only in tropical and subtropical endemic areas to an infection that now appears globally, with an observed 30-fold increase in infections. More than 2.5 billion people (more than 40% of world’s population) are currently at risk of DENV infection [2]. The World Health Organization (WHO) estimated the incidence of DENV infection to be 50–100 million annually, with approximately 500,000 severe cases (mostly in children) that require hospitalization [2]. The mortality rate among severe cases of DENV disease, which include dengue hemorrhagic fever (DHF) and dengue shock syndrome (DSS), is about 2.5%.

For decades, attempts have been made to develop vaccines to prevent DENV infection. Major obstacles to vaccine development include antibody-dependent enhancement (ADE) and original antigenic sin, which are caused by over-response of the immune system in secondary DENV infection via cross-reactive antibodies (at the subneutralizing level) and cross-reactive T cells from previous infection [3]. Thus, immunization by vaccine is required to fully protect a person from the four circulating DENV serotypes. A tetravalent vaccine (Dengvaxia^®^ (CYD-TDV) Sanofi Pasteur, Lyon, France) was approved by the U.S. Food and Drug Administration (FDA) and has been licensed in 20 countries [4]. However, the clinical data revealed that this vaccine confers partial protection only against DENV2 infection [5]. Moreover, there are some safety concerns since this vaccine was reported to be associated with increased incidence of severe DENV disease in children aged younger than 9 years [6]. Therefore, a safer, more effective, and more comprehensive vaccine is required. In addition to the aforementioned deficiencies in DENV prevention, no specific drugs have been developed to treat DENV infection, alleviate disease severity, or reduce the risk of death from severe DENV disease.

The positive association between viral load and clinical severity suggested the possibility of using anti-DENV drugs to reduce the risk of severe complications in patients with DENV infection [7,8]. To that end, there have been intensive efforts to discover and develop anti-DENV drugs to treat DENV infection. Several types of anti-DENV agents that disrupt the virus life cycle have been reported. Two categories of anti-DENV agents (according to the protein targets) have been intensively investigated by several researchers. The first category comprises anti-DENV agents that inhibit the key virus proteins involved in virus entry/replication, including the structural proteins envelope (E) and membrane (prM/M) [9]; and, the non-structural proteins NS1, NS2B-NS3 proteinase, NS4A, NS4B, and NS5 [10]. The second category includes anti-DENV agents that interrupt the functions of host proteins that play a role in the virus life cycle, including those that affect viral receptors, host metabolic processes, host cell immunity, and autophagy [11]. Several research groups, including ours, have recently been working to identify anti-DENV inhibitors. Many types of inhibitors have been reported, and the efficiencies of some anti-DENV agents have also been demonstrated. Our research group previously reported peptide inhibitors targeting DENV E protein [12,13,14], vivo-morpholinol targeting DENV genome [15,16], and small compound inhibitors (i.e., compound A) targeting critical host proteins [17,18,19]. Although various antiviral agents were shown to significantly inhibit DENV infection in vitro or in animal models, none of these agents have yet been approved for treatment of the DENV infection.

Medicinal plants that are widely used in traditional medicine are attractive sources of bioactive proteins and peptides that demonstrate a broad spectrum of activities, including anti-inflammation, antimicrobial, and antioxidant actions. Consistent with that, the Thai medicinal plants that comprise Thai traditional medicine contain peptides of interest that have been and are being investigated by our group in an attempt to identify novel peptide inhibitors to treat DENV infection. We recently reported novel peptide inhibitors isolated from *Acacia catechu* extract [14]. The identified peptide inhibitors were shown to have broad anti-DENV activities that work to effectively inhibit all four DENV serotypes at micromolar levels [14]. In the present study, anti-DENV peptide inhibitors were further characterized and examined for their inhibitory mechanism. That investigation revealed the most effective peptide to be from *Acacia catechu* extract (designated Pep-RTYM), and this peptide demonstrated direct interaction with DENV particles. Time-of-addition study demonstrated that Pep-RTYM interacted with DENV particles and inhibited cell entry, and inhibited DENV replication and production in a dose-dependent manner. This finding suggests a potential role of Pep-RTYM in the development of a novel anti-DENV drug.

## 2. Materials and Methods

### 2.1. Synthetic Peptides, Cell Lines, and Virus Propagation

**Figure 1:**
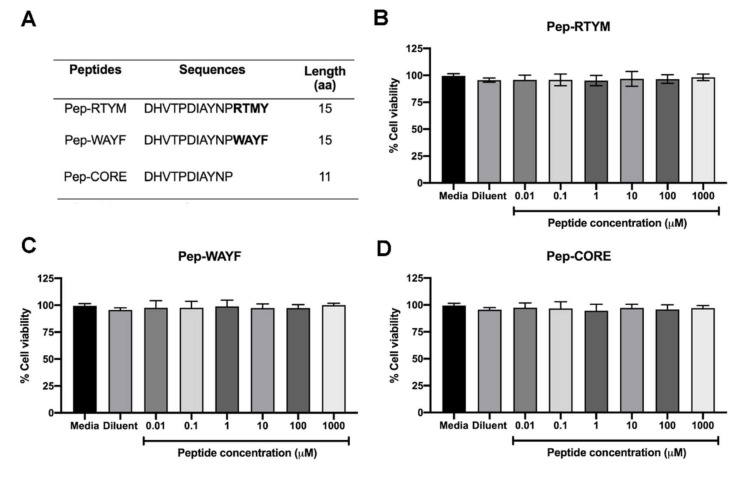
Cytotoxicity of bioactive peptide inhibitors. Three bioactive peptides derived from *Acacia catechu* were determined for their toxicity to Vero cells (**A**). Cell viability after 48 h treatment of Pep-RTYM (**B**), Pep-WAYF (**C**), and Pep-CORE (**D**) was represented as percentage (%) of cell viability relative to that of non-treatment control, which was set as 100% cell viability.

Peptides derived from *Acacia catechu* extract were synthesized by custom order (Selleck Chemicals, Houston, TX, USA). Three synthetic peptides, which were designated Pep-RTYM, Pep-WAYF, and Pep-CORE, shared the same 11 amino acid sequences at their N-termini. The first two peptides are 15 amino acids in length, and the third is 11 amino acids in length (Figure 1A). A negative peptide that was designated Pep-Scr was synthesized that contained the amino acid composition of Pep-RTYM, but the amino acid sequence was randomly rearranged, and this peptide was used as the negative control (Figure 1A). Vero cells (kidney epithelial cells isolated from African green monkeys) were cultured in minimal essential medium (MEM), and Huh7 cells (hepatocellular carcinoma cell line) were grown in Dulbecco’s modified Eagle medium/nutrient mixture F-12 (DMEM/F-12) supplemented with 10% (*v*/*v*) fetal bovine serum (FBS), 2 mM glutamine, and antibiotics at 37 °C with 5% CO_2_. The propagation of DENV serotype 2 (DENV2) strain 16681 was performed using C6/36 cells. Culture supernatant containing DENV was stored at −70 °C until use.

### 2.2. Cell Viability

The cell viability was conducted to examine the toxicity of synthetic peptides to the cells. Briefly, Vero cells were plated in 96-well format (2 × 104 cells per well) and cultured overnight. At the time of experiment, three peptide Pep-RTYM, Pep-WAYF, and Pep-CORE were prepared in 10-fold serial dilutions ranging from 0.01–1000 μM and added into the cultured cells (100 μL/well). After 48 h of incubation, the cell viability was measured by using the PrestoBlue™ cell viability reagent (Thermo Fisher Scientific, Waltham, MA, USA). The color changes of reagent representing the reducing viability of the cultured cells were measured by monitoring the absorbance at 570 nm and the absorbance at 600 nm was used as a reference wavelength. The cell viability was calculated as percentage of the total cell viability (% cell viability) relative to that of nontreated control as the following equation:

% cell viability = [(OD570^treated cell^ − OD600^treated cell^)/(OD570^control cell^ − OD600^control cell^)] × 100

### 2.3. Focus-Forming Unit (FFU) Assay

Briefly, Vero cells were plated in 96-well format (2 × 104 cells per well) for overnight. At the time of experiment, the DENV was prepared in serial dilution (10-fold serial dilution) and infected to Vero cells. The media was then replaced with 2% carboxymethylcellulose medium. The infected cells were detected after 72 h of infection. The foci were stained by using 4G2 antibody (antibody targeting DENV envelope protein) followed by rabbit anti-mouse IgG antibody horseradish peroxidase (HRP) conjugate (Dako, Glostrup, Denmark). The substrate 3,3′-diaminobenzidine (DAB) substrate (Sigma-Aldrich, St. Louis, MO, USA) was added to the well. The focus formation was visualized and counted under a light microscope (Nikon Instruments, Tokyo, Japan).

### 2.4. Inhibition of DENV Focus Formation

Vero cells were plated in 96-well format (2 × 104 cells per well) for overnight. The cells were infected with 400 FFU of DENV in the presence or absence of custom synthesized peptides (Selleck Chemicals) at the indicated concentrations for 2 h. The media was then replaced with 2% carboxymethylcellulose medium. The numbers of FFUs were determined and counted manually under a microscope, as previously described. The percentage of inhibition was calculated relative to that of the infected control with no peptide treatment (set as 0% inhibition). Half-maximal inhibitory concentration (IC50) was calculated using non-linear regression in GraphPad Prism version 8 (GraphPad Software, Inc., San Diego, CA, USA).

### 2.5. Inhibition of DENV Infection and Production

Immunofluorescence assay (IFA) was performed to examine the reduction of DENV infectivity. Vero cells were seeded in a 24-well plate (1 × 10^5^ cells per well) for overnight. The cells were infected with approximately 4 × 10^4^ FFU/mL of DENV in the presence or absence of custom synthesized peptides for 2 h. Then the culture medium was replaced with fresh medium. At 72 h after infection, infected cells and culture supernatant were collected. Monoclonal 4G2 antibody was then added to cells, followed by Alexa Fluor 488 goat anti-mouse IgG (Invitrogen, Carlsbad, CA, USA). The nuclei were stained with Hoechst^®^ 33,342 nucleic acid stain (Invitrogen, Carlsbad, CA, USA). The infected cells were visualized using the laser scanning confocal microscope (LSM800, Zeiss, Jena, Germany). The viral titer in the collected culture supernatant was determined and counted using FFU assay as previously described.

To determine the efficiency of pep-RTYM in Huh7 cells, the cells were plated in a 24-well plate (1 × 10^5^ cells per well) and cultured overnight. The cultured cells were infected with 1 × 10^5^ FFU/mL of DENV in the presence or absence of custom synthesized peptides for 2 h. The culture medium was replaced with fresh medium and further incubated for 48 h. The IFAs were performed as described above 

### 2.6. Time-of-Addition and Peptide-Virus Binding Assays

The inhibitory mechanism of synthetic bioactive peptides was investigated by time-of-addition assay. Briefly, Vero cells were seeded at 4 × 10^4^ cells per well in a 24-well plate the day before the experiments. For pre-incubation condition, DENV (1 × 10^5^ FFU/mL) and 25 μM of synthetic bioactive peptides were incubated for 30 min at 37 °C before being added to Vero cells. For co-infection and post-infection, 25 μM of synthetic bioactive peptides were added to Vero cells together with DENV, and after 2 h of DENV infection, respectively. The viral titer in the collected culture supernatant was determined and counted using FFU assay, as mentioned before in Section 2.3.

### 2.7. Quantitative Reverse Transcription Real-Time PCR (qRT-PCR)

To investigate the inhibitory effect of synthetic bioactive peptide on the viral entry step, qRT-PCR was performed. Briefly, Vero cells were seeded at 1 × 10^5^ cells per well in a 24-well plate for 24 h before the experiments. DENV2 of approximately 4 × 10^4^ FFU/mL was added in the presence or absence of Pep-RTYM or irrelevant negative peptide (Pep-Scr) (Selleck Chemicals). Cells were incubated for 2 h at 37 °C with 5% CO_2_. The cells were washed three time with cold PBS to remove the unbound viruses. The amounts of internalized viruses were measured by using real-time PCR to detect the viral genomic RNA. The total RNA was extracted from the infected cells by using TRIzol™ reagent (Invitrogen, Carlsbad, CA, USA). The cDNA synthesized was performed using a cDNA synthesis kit and taking 500 ng RNA as the template (Toyobo Life Science, Osaka, Japan). Real-time PCR was performed using specific primers (Table S1) and LightCycler 480 SYBR Green I Master Mix (Roche Diagnostics, Mannheim, Germany). The cycle threshold (Ct) data were shown in Table S2 and analyzed for relative quantification of intracellular virus relative to that of house-keeping gene (GAPDH), and the results are shown as fold changes of which the infection control was set as 1.

### 2.8. Virus Binding Assay

Direct interaction of Pep-RTYM to DENV was determined by using enzyme-linked immunosorbent assay (ELISA). The C6/36 culture supernatant containing DENV was prepared in carbonate-bicarbonate buffer (pH 9.6) and coated onto Nunc MaxiSorp™ high protein-binding capacity 96-well ELISA plates (Thermo Fisher Scientific, Waltham, MA, USA) overnight before the day of experiment. In addition, culture supernatant of C6/36 without the virus (called mock control) was coated onto the plate and used as the negative control for the system. The plate was then blocked with 1% BSA (*w*/*v*) at room temperature for one hour. Biotinylated synthetic biotin-conjugated Pep-RTYM was prepared at the indicated concentration and added to the plate for 30 min at 37 °C. Horseradish peroxidase conjugated streptavidin (Thermo Fisher Scientific) was added for 30 min and then washed with PBS containing 0.1% Tween-20 3 times before adding TMB (3,3,5,5-tetramethylbenzidine) substrate (Invitrogen, Carlsbad, CA, USA). Absorbance at optical density (OD) 650 was measured at the indicated time points.

### 2.9. Molecular Docking

The sequences of peptides were created by Discovery Studio 2018 [20]. The peptide substrate was saved as a mol2 format as a ligand input for GOLD docking program Version 2020.1 [21]. The structure of DENV2 envelope (E) protein was used as a protein target (PBD ID:1OAN) [22]. The GOLD docking was performed as a standard protocol with GOLDscore by setting the binding site on domain III of the E protein, which was reported to be bound by neutralizing antibody [23]. The best docking pose was chosen for further analysis by Discovery Studio 2018.

### 2.10. Statistical Analysis

Mean and standard error of the mean (SEM) were analyzed via GraphPad Prism Software version 8 (GraphPad Software, Inc., San Diego, CA, USA) using data from three independent experiments. Comparisons to identify statistically significant differences were performed using Student’s *t*-test. Asterisks were used to indicate different levels of statistical significance, as follows: * *p* < 0.05; ** *p* < 0.01, *** *p* < 0.001, and **** *p* < 0.0001.

## 3. Results

### 3.1. Synthetic Bioactive Peptide Inhibited DENV Infection

Our research group previously reported that bioactive peptides extracted from *Acacia catechu* extract could effectively inhibit DENV infection [14]. Two bioactive peptides (both 15 amino acids in length) that share 11 amino acids at the N-terminus (designated Pep-RTYM and Pep-WAYF) were identified. In this study, the efficiencies of the synthetic bioactive peptides Pep-RTYM and Pep-WAYF, in addition to Pep-CORE, all sharing 11 amino acids at their N-termini (Figure 1A), were compared and analyzed for their IC50 values relative to their ability to inhibit DENV focus formation. The cytotoxicity of these 3 synthetic peptides on cell viability was initially examined. Treatment with synthetic peptides up to 1 mM caused only slight effects on cell viability (greater than 85% cell viability compared to that of the no treatment control), which suggested that synthetic peptides had very low cytotoxic effect on Vero cells (Figure 1B–D). Non-toxic concentrations of synthetic peptides were used for all subsequent experiments.

**Figure 2:**
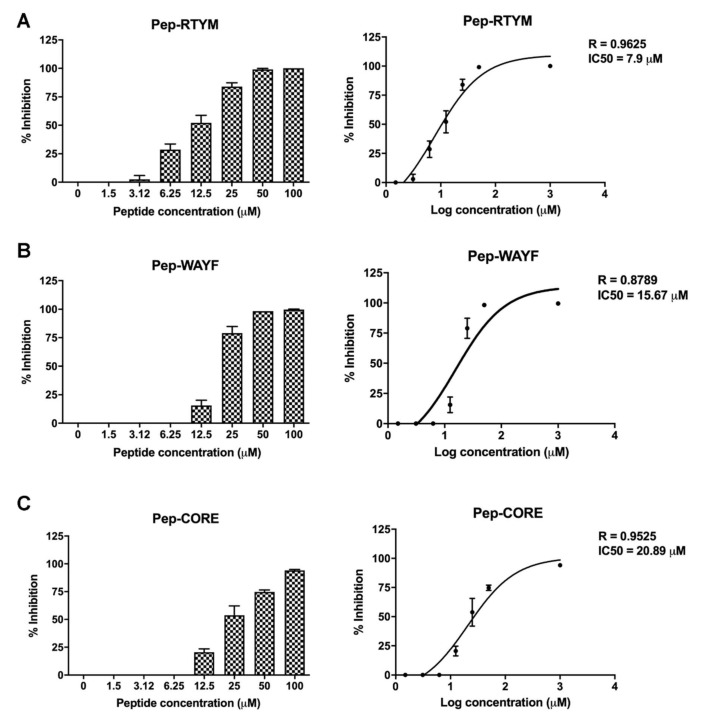
The efficiencies of bioactive peptide inhibitors on dengue virus (DENV) infection. The efficiencies of peptide inhibitors to inhibit DENV2 infection in Vero cells was determined. DENV2 was treated with various concentrations of the peptide inhibitors Pep-RTYM, Pep-WAYF, or Pep-CORE prior to virus infection. Reduction of focus forming units was determined, and the IC50 value for Pep-RTYM (**A**), Pep-WAYF (**B**), and Pep-CORE (**C**) was determined by non-linear regression analysis.

Synthetic peptides at concentrations of 1.5–100 μM were used to examine inhibitory activities against DENV2 by FFU-reduction assay. Treatment with Pep-RTYM, Pep-WAYF, or Pep-CORE caused a reduction of focus formation in a dose-dependent manner (Figure 2A–C). The IC50, as analyzed by non-linear regression, revealed that Pep-RTYM had the best inhibitory activity with an IC50 of 7.9 μM (Figure 2A), which was more effective than those of Pep-WAYF (IC50: 15.67 μM) and Pep-CORE (IC50: 20.89 μM) (Figure 2B,C). These results suggest the importance of the amino acid sequences at the N-terminus, and also their differences at the C-terminus of synthetic peptides relative to their effect on inhibitory activities.

### 3.2. Pep-RTYM Efficiently Inhibited DENV Production at the Early Steps of Infection

**Figure 3:**
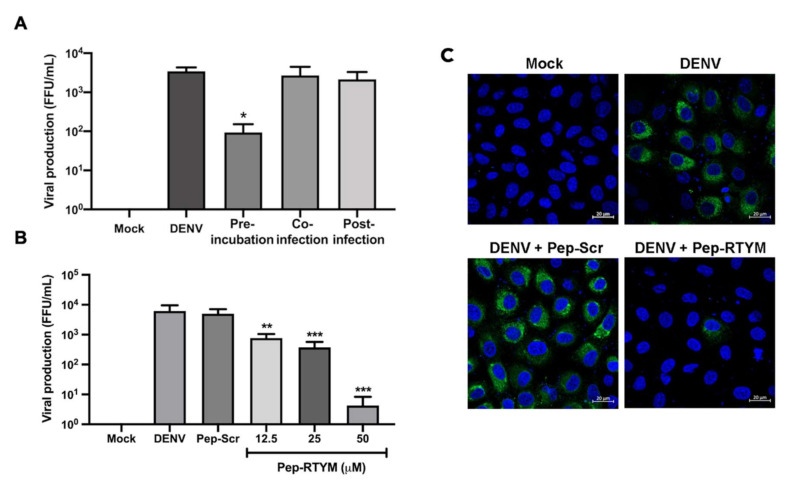
Inhibitory effect of Pep-RTYM peptide inhibitor on DENV infection in Vero cells. DENV2 was treated with 25 μM of Pep-RTYM prior to virus infection (pre-incubation), during virus infection (co-infection), or upon virus infection (post-infection) to investigate the effect of this peptide on virus replication (**A**). The inhibitory effect of Pep-RTYM was shown to lower virus production (**B**) and the number of infected cells (**C**) compared to the irrelevant negative peptide (Pep-Scr) using virus titration and immunofluorescence assay, respectively. Asterisks were used to indicate different levels of statistical significance, as follows: * *p* < 0.05; ** *p* < 0.01, and *** *p* < 0.001.

To characterize the inhibitory effect of Pep-RTYM on DENV, time-of-addition assay was performed to examine the effect of this peptide on the dengue life cycle. In pre-treatment condition, where the peptide and virus interacted prior to cellular infection, the result showed that Pep-RTYM greatly inhibited DENV infection (Figure 3A). When Pep-RTYM treatment was administered at the same time as cellular infection (co-infection) or after cellular infection (post-infection), the peptide failed to inhibit virus infection (Figure 3A). Our result revealed that Pep-RTYM could effectively inhibit DENV infection in the early step of infection, and this might be related to the antiviral mechanism of this peptide.

To re-examine the inhibitory effects of Pep-RTYM on DENV production and infection, 12.5, 25, and 50 μM of Pep-RTYM was added to DENV2 before infection in Vero cells. After that, the number of DENV in cell culture supernatant was determined by FFU method, and in DENV2-infected Vero cells by IFA. The results showed that Pep-RTYM caused significant reduction in DENV2 production in cell culture supernatant (Figure 3B). Treatment with Pep-RTYM at a concentration of 50 μM could inhibit DENV2 production more than 1000-fold compared to that of Pep-Scr. Similarly, the numbers of infected Vero cells examined by intracellular DENV E protein staining were markedly reduced after Pep-RTYM treatment (Figure 3C).

### 3.3. Pep-RTYM Binds to DENV Particles

**Figure 4:**
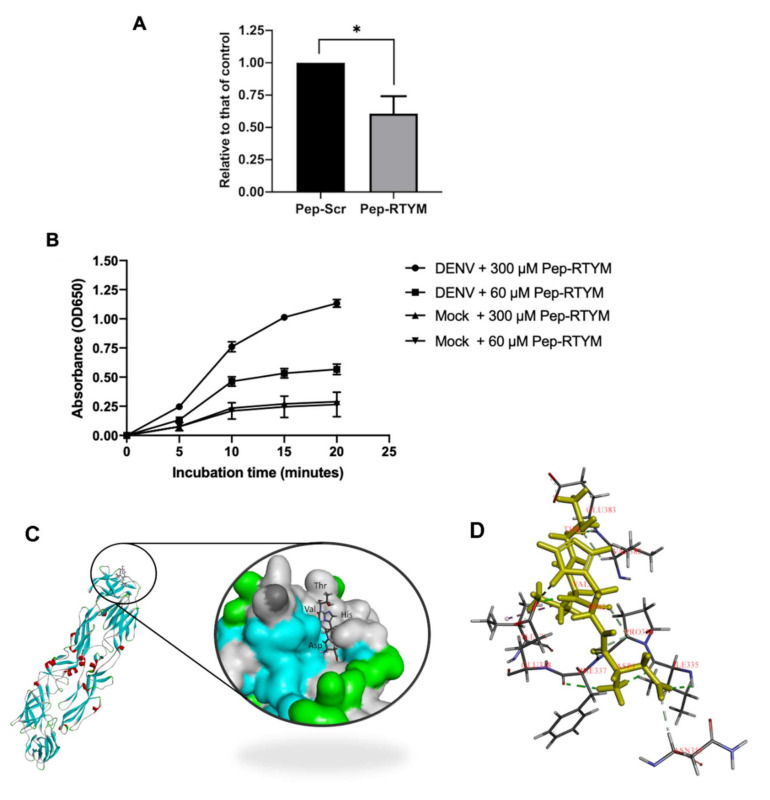
Mechanism of peptide inhibitor on inhibiting DENV infection. The ability of Pep-RTYM to inhibit virus entry was determined using real-time quantitative reverse transcription polymerase chain reactions. The internalized virus particles were measured in DENV-infected cells with 25 μM Pep-RTYM treatment compared to that of Pep-Scr treatment (**A**). The dose-dependent capacity of Pep-RTYM to directly interact to the virus particle was analyzed by peptide-virus binding assay (**B**). The molecular docking showed the interaction of Pep-RTYM (yellow) to DENV EDIII protein (**C**). The predicted biding residues of Pep-RTYM (yellow) and EDIII were labeled (**D**). Asterisks were used to indicate different levels of statistical significance, as follows: * *p* < 0.05.

Pep-RTYM was shown to inhibit DENV infection in the early step of infection based on the results of time-of-addition assay. Since the entry step is critical during the early phase of infection, it is possible that Pep-RTYM inhibited the virus entry step by disrupting binding of the virus to the host cell receptor. To investigate whether peptide function through virus entry inhibition, a number of internalized virus was measured by using qRT-PCR to determine viral genomic RNA after 2 h of infection, comparing between the infected with Pep-Scr and Pep-RTYM treatment at equal concentrations. Consistent with the results of time-of-addition assay, virus internalization was significantly reduced in the presence of 25 μM pep-RTYM (approximately 40% reduction) (Figure 4A).

Virus-receptor binding is a crucial step for virus entry, and binding of our peptide to the virus particle might disturb this critical step. To investigate the possibility that Pep-RTYM could bind to the virus particle, we performed a virus binding assay. The signal intensity of Pep-RTYM binding to DENV particles was compared to that of negative mock control. Binding of Pep-RTYM to DENV showed a strong binding signal compared to that of mock control in a dose- and time-dependent manner (Figure 4B). This result suggests that Pep-RTYM could bind to DENV, which appears to disrupt the interaction between the virus and the host receptor leading to inhibition of infection.

To illustrate the ability of Pep-RTYM to inhibit DENV entry, molecular docking was performed to visualize the potential binding of peptide to DENV2 envelope (E) protein. The peptides were shortened to allow sampling of most of the potential rotational bonds in the peptide. The result revealed the interaction of the amino acids DHVT of the peptide stably interacted to E protein via several hydrogen bonds; Asp1 with Ile335 and Asn355; His2 with Glu333 and Pro336; Thr4 with Val382 and Glu383 (Figure 4C).

### 3.4. Inhibitory Effect of Pep-RTYM on DENV Infection in Hepatocytes

**Figure 5:**
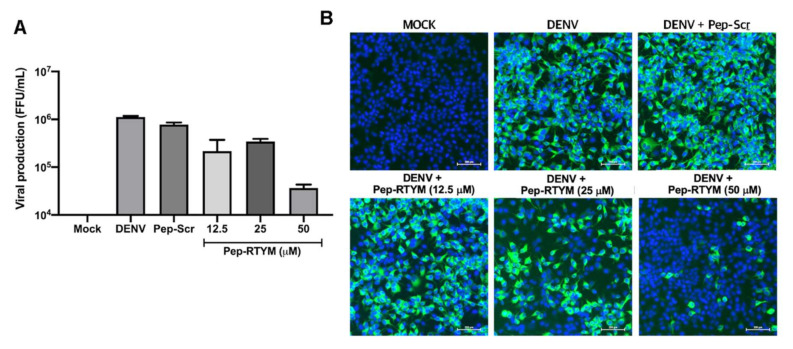
Inhibitory effect of Pep-RTYM on DENV infection in infected hepatocytes. DENV2 was treated with Pep-RTYM at concentrations of 12.5, 25, or 50 μM prior to virus infection in Huh7 cells. The inhibitory effect of Pep-RTYM was determined at 48 h after infection by analysis of the level of decreased virus production (**A**) and the number of infected cells (**B**) compared to that of Pep-Scr using virus titration and immunofluorescence assay, respectively.

Since Pep-RTYM directly inhibited the DENV particle, we hypothesized that Pep-RTYM could broadly protect various target cell types from DENV infection. Thus, the antiviral activity of Pep-RTYM was confirmed for its therapeutic potential to inhibit virus infection in human cells. Hepatocytes are well documented as a critical target of DENV infection, and hepatocyte injury was reported as a hallmark of severe DENV infection [24]. We thus determined the antiviral effect of Pep-RTYM for reducing viral production and the number of infected cells in hepatocyte Huh7 cells. The viruses were treated with Pep-RTYM prior infection of Huh7 cells. The cytotoxicity of Pep-RTYM were firstly determined in Huh7 cells (Figure S1). The viruses were then treated with sub-lethal doses of Pep-RTYM prior to the infection of Huh7 cells. The production of new DENV progenies was investigated after 48 h of infection. The result revealed virus production in Pep-RTYM treated cells to be greatly reduced in a dose-dependent manner (Figure 5A). Treatment of DENV with 50 µM Pep-RTYM decreased viral production 10-fold compared to that of the untreated control. IFA confirmed that Pep-RTYM treatment reduced the number of infected cells (Figure 5B), which revealed the potential of Pep-RTYM for DENV infection inhibition in human cells.

## 4. Discussion

Therapeutic peptides represent an attractive alternative for development into anti-DENV drugs due to their safety and their diverse biological and chemical properties. Many new therapeutic peptides have been developed and reported [25]. The development of over 400 therapeutic peptides was reported in 2017, and 68 of those were approved for use as therapeutic peptides. The published evidence demonstrates the feasibility of peptide-based drugs [25]. Peptides can be used as an antiviral agent to inhibit virus replication by either directly inhibiting the virus life cycle or by manipulating host response. A peptide inhibitor targeting HIV (Enfuvirtide (T20)) was approved for HIV/AIDS treatment, which supports the feasibility of peptides as antiviral agents. However, there is currently no approved peptide inhibitor for treatment of DENV infection. Our group recently reported peptide inhibitors isolated from *Acacia catechu* extract that effectively inhibited DENV infection. Treatment with these bioactive peptide inhibitors could broadly inhibit all serotypes of DENV [14]. To develop bioactive peptide inhibitors as antiviral agents for DENV infection treatment, we defined the inhibitory mechanism and the efficiencies of bioactive peptides for inhibiting DENV infection in human hepatocyte cells.

The bioactive peptide Pep-RTYM was found to be the most efficient peptide for significantly inhibiting the early step of DENV infection according to time-of-addition assay. The results showed that incubation of Pep-RTYM with the virus prior to infection (pre-incubation) provided the most effective inhibition, whereas peptide treatment after DENV infection (post-infection) failed to achieve inhibition effect (Figure 3A). Furthermore, our virus binding assay demonstrated that Pep-RTYM could directly interact with virus particles in a dose-dependent manner (Figure 4A), which suggests that the inhibitory mechanism of our peptide required binding of the peptide to the virus with subsequent inhibition of cell entry during the early step of infection.

Entry of DENV into host cells is a key step during the early step of infection, and entry is mediated via well-orchestrated events specific to DENV envelope protein function. Envelope (E) protein, which is 495 amino acids in length, is comprised of three distinct domains (DI, DII, and DIII) that are responsible for virus binding to host cell surface, fusion, and virus internalization [26]. Interruption of one of these processes could influence the inhibition of DENV entry. Our real-time RT-PCR results showed that Pep-RTYM treatment resulted in a significantly lower amount of viral genomic RNA entering the host cells (Figure 4A). This result appears to suggest that the biding of Pep-RTYM to virus particles might disrupt the ability of the virus to bind to the host cell receptor or possibly block the internalization of the virus into the host cells.

DENV EDIII, which is an immunoglobulin-like carboxy terminal domain that is responsible for virus-host receptor binding, is one of the important target sites for blocking virus entry. Binding of inhibitors to the binding site of EDIII would physically hide that site, which would result in failure of the virus to bind to the host cell receptor. Several heparan sulfate mimetics to target EDIII of DENV have been reported, and activities and effects demonstrating inhibition of DENV infection were observed [27,28,29]. In the present study, Pep-RTYM, which could bind to DENV and exert inhibitory effect independent of host cell type, might act via prevention of EDIII to attach to the host receptor. Treatment of Pep-RTYM could significantly decrease DENV production in Vero-kidney epithelial cells and Huh7 hepatocyte cells but the effect was lower in Huh7 cells (40-fold reduction) compared to Vero cells (1000-fold reduction), which might be due to the different natures of their receptors. The binding of peptide might interfere with the virus-host receptor interaction that varied because different protein receptors might be used in different cell types. To examine the binding position of Pep-RTYM to EDIII protein, we conducted the molecular docking to visualize the potential binding of this peptide to DENV2 envelope protein by using GOLD docking program Version 2020.1. The result showed that Pep-RTYM interacts with several amino acids of EDIII, which supports this hypothesis (Figure 4C). Pep-RTYM was predicted to interact with the amino acids at the positions: I335, P336, F337, E338, V382, and E383. Interestingly, this region is highly conserved among DENV serotypes (Figure S2). Previously, we have shown the antiviral activity of *Acacia catechu* extract against all four serotypes of DENV [14]. This inhibitory effect would be the result of the interaction of pep-RTYM to the conserved amino acid residues of DENV E protein.

Another possible mechanism by which a peptide inhibitor inhibits attachment of the virus to the host cell is via induction of conformational change in virus structure. Several peptides that inhibit DENV infection were previously reported, and those peptides caused structural abnormality and alteration of E protein arrangement [13,30]. However, the structure abnormality of DENV that is caused upon Pep-RTYM treatment needs to be further investigated by standard or cryogenic electron microscopy. Since Pep-RTYM acts via targeting the early step of DENV infection, theoretically therapeutic strategy is to deliver peptide prior to the virus entry into the host cells. In reality, DENV infection is a dynamic event; the new progeny of DENV can spread from the site of infection into the blood stream resulting in systemic infection and the viruses can subsequently infect other cells and organs in the body. Accordingly, peptide inhibitor can be delivered to the patients in the early phase of infection or during viremia. If peptide inhibitor was appropriately maintained in blood stream, it would target those new generations of viruses to control the infection rate, limit the infection area, and lower the risk of severe infection. The treatment of peptide inhibitor after the viremia phase is possible, although the treatment at the early phase of infection should be more effective. On the other hand, Pep-RTYM would not be suitable to use for prevention since our pre-treatment assay demonstrated peptide failed to protect the cells from the virus. The right timing is therefore critical as the key success factor of peptide therapeutic strategy. To enhance the possibility of Pep-RTYM treatment, the rapid screening test of DENV infection, especially during the epidermis and outbreak, is necessary to monitor the disease stage and evaluate the peptide treatment benefit.

Low stability and the bioavailability of peptides are the other major concerns when developing a peptide inhibitor as a therapeutic agent since an unmodified peptide is rapidly degraded by proteases in the human body. Although the oral administration is preferable, peptide/protein drugs available in the market mostly are therapeutically delivered via intravenous (iv) or subcutaneous injection to avoid the gastrointestinal degradation. As the consequence of peptide degradation by gastrointestinal proteases and low absorption due to the epithelial barrier and efflux pump [31], it was estimated that only 2% of peptide/protein remained after oral administration. Furthermore, the peptides/proteins will encounter systemic proteases that reduce their stabilities. Therefore, absorption enhancer, stability enhancer, and carrier system are critical issues to solve these problems. Previously, it was reported that the instability of peptides may be possible to overcome via chemical modification [32]. Cyclic-peptide [33], D-amino acid [34], chemical modification by C-amidation [33], N-acetylation [33], and -amino-3-guandino-propionic acid (Agp) replacement [35] were all reported to be alternative strategies for successfully increasing the half-life of peptides. These technological advancements will further facilitate the development of peptide inhibitors that can be used as therapeutic drugs. An effective vector control, protective vaccine, and strong antiviral inhibitors would offer vastly improved results in the fight against DENV infection in endemic countries.

## 5. Conclusions

The synthetic bioactive peptide Pep-RTYM, which was isolated from *Acacia catechu* extract, interacted with DENV particles and inhibited cell entry, and inhibited DENV replication and production in a dose-dependent manner. This finding suggests a potential role of Pep-RTYM in the development of a novel anti-DENV drug.

## Supplementary Materials

The following are available online at https://www.mdpi.com/1999-4915/12/11/1267/s1, Table S1: Primer sequences for real-time PCR; Table S2: The ability of Pep-RTYM to inhibit virus entry by real-time PCR; Figure S1: Cytotoxicity of bioactive peptide inhibitors in Huh7 cells; Figure S2: DENV envelope protein sequence alignment.
