# Supplementary material for: A Synthetic Bioactive Peptide Derived from the Asian Medicinal Plant Acacia catechu Binds to Dengue Virus and Inhibits Cell Entry
Source: Viruses. 2020 Nov 6;12(11):1267. doi: 10.3390/v12111267 (PMC7694746; doi:10.3390/v12111267)

Supplementary Table S1 Primer sequences for real-time PCR

No.	Name	Sequence (5’ to 3’)	Length (bases)	Reference
1	D2R	CCGGCTCTACTCCTATGATG	20	[1]
2	D2L	ATCCAGATGTCATCAGGAAAC	21	[1]
3	GAPDH-R	CGACCACTTTGTCAAGCTCA	20	[2]
4	GAPDH-L	AGGGGTCTACATGGCAACTG	20	[2]

References

1. Yenchitsomanus P.T., Sricharoen P., Jaruthasana I., Pattanakitsakul S.N., Nitayaphan S., Mongkolsapaya J., Malasit P. (1996) Rapid detection and identification of dengue viruses by polymerase chain reaction (PCR). Southeast Asian J Trop Med Public Health;27:228–236.
2. Panya A, Sawasdee N, Srisawat C, Yenchitsomanus P, Peerapittayamongkol C. Expression of zinc finger and homeobox 2 in erythroleukemic cells and gamma-globin expression. ScienceAsia 2010;36:342-5

Supplement Table S2 The ability of Pep-RTYM to inhibit virus entry by real-time PCR. The internalized virus particles were measured in DENV-infected cells using real-time quantitative reverse transcription polymerase chain reactions.

Samples	Ct^{GAPDH}	Ct^{DENV}	Delta Ct (Ct^{GAPDH} -Ct^{DENV})
Nontreatment control	26.87±1.19	28.55±0.55	1.68±0.65
Pep-RTYM treated cells	26.65±0.27	29.12±0.26	2.47±0.33
Mock	28.00±0.85	-	-

Supplementary Figure S1 Cytotoxicity of bioactive peptide inhibitors in Huh7 cells. The Pep-RTYM were determined for their toxicity to Huh7 by determining cell viability after 48 hours of peptide treatment. The cell viability was represented as percentage (%) of cell viability relative to that of non-treatment control, which was set as 100% cell viability.

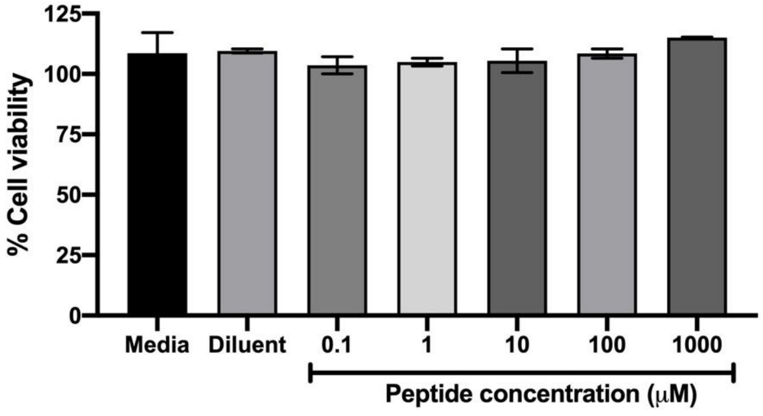

Supplementary Figure S2 DENV envelope protein sequence alignment. The amino acid sequences of DENV envelope proteins including DENV1 (strain Hawaii), DENV2 (strain 16681), DENV3 (strain H87), and DENV4 (strain H241) were aligned by using the Bioedit program. The predicted interacting residues of Pep-RTYM was indicated with red box.

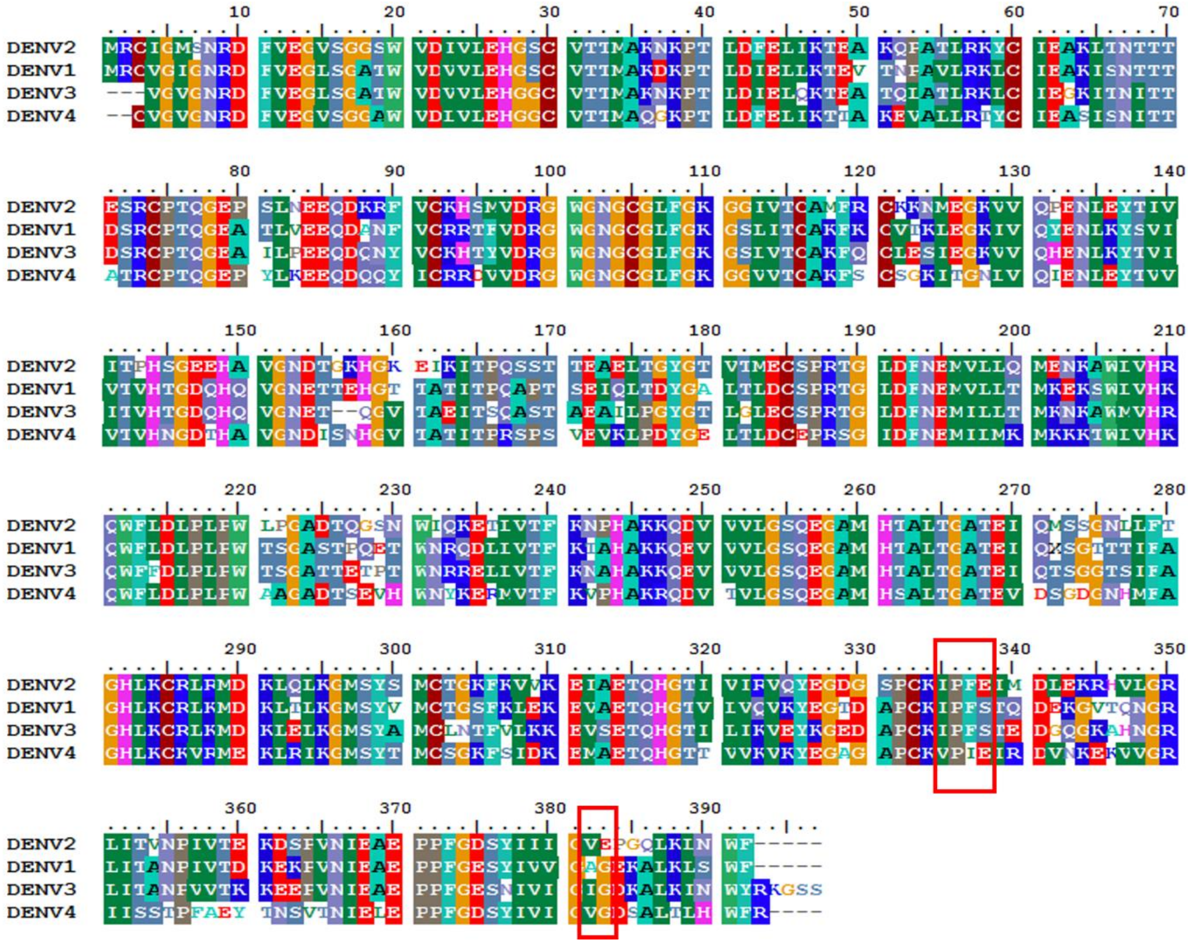

Supplement: Supplementary file 1 [file viruses-12-01267-s001.pdf]
